# Photon *Versus* Proton Beam Therapy for T1–3 Squamous Cell Carcinoma of the Thoracic Esophagus Without Lymph Node Metastasis

**DOI:** 10.3389/fonc.2021.699172

**Published:** 2021-06-21

**Authors:** Yang-Gun Suh, Unurjargal Bayasgalan, Heung Tae Kim, Jong Mog Lee, Moon Soo Kim, Youngjoo Lee, Doo Yeul Lee, Sung Uk Lee, Tae Hyun Kim, Sung Ho Moon

**Affiliations:** ^1^ Proton Therapy Center, Research Institute and Hospital, National Cancer Center, Goyang, South Korea; ^2^ Department of Radiation Oncology, National Cancer Center, Ulaanbaatar, Mongolia; ^3^ Department of Internal Medicine, Research Institute and Hospital, National Cancer Center, Goyang, South Korea; ^4^ Department of Thoracic Surgery, Research Institute and Hospital, National Cancer Center, Goyang, South Korea

**Keywords:** esophageal cancer, proton beam therapy, 3-dimensional radiotherapy, intensity-modulated radiotherapy, endoscopic submucosal dissection, lymph node metastasis, organs at risk

## Abstract

**Background and Purpose:**

We compared treatment outcomes and toxicities of photon radiotherapy *versus* proton beam therapy (PBT) and evaluated radiation field effects for T1–3 squamous cell carcinoma of the thoracic esophagus (EC) without lymph node metastasis.

**Methods:**

Medical records of 77 patients with T1–3N0M0 thoracic EC treated with radiotherapy between 2011 and 2019 were retrospectively analyzed. Among these patients, 61 (79.2%) individuals had T1 EC. The initial clinical target volume encompassed the whole esophagus with or without supraclavicular and/or abdominal lymph nodes (extended-field radiotherapy; 67 patients, 87.0%) or the area 3–5 cm craniocaudally and 1–2 cm radially from the gross tumor volume (involved-field radiotherapy; 10 patients, 13.0%). The final clinical target volume included margins of at least 1 cm from the gross tumor volume, with total radiation doses of 50–66 (median, 66) cobalt gray equivalent. Three-dimensional conformal radiotherapy, intensity-modulated radiotherapy, and PBT were used in twenty-four, five, and forty-eight patients, respectively. Concurrent chemotherapy was administered to 17 (22.0%) patients overall and only five (8.0%) T1 patients.

**Results:**

PBT showed significantly lower lung and heart radiation exposure in mean dose, V5, V10, V20, and V30 than photon radiotherapy. The median follow-up for all patients was 46 (interquartile range, 22–72) months. The 5-year progression-free survival and overall survival rates were 56.5 and 64.9%, respectively, with no significant survival difference between photon radiotherapy and PBT. In patients with T1 EC, 5-year progression-free survival and overall survival rates were 62.6 and 73.5%, respectively.

**Conclusions:**

Extended-field radiotherapy using modern radiotherapy techniques without chemotherapy showed satisfactory clinical outcomes for lymph node-negative T1 EC.

## Introduction

The esophagus has abundant and complex lymphatic channels that begin from the submucosal layer and the outer layers differently communicating intramurally and extramurally (1). Therefore, lymph node (LN) metastasis frequently develops after treatment of esophageal cancer (EC), even in clinically LN-negative T1 superficial EC. Nodal skip metastasis, which is found far from the original tumor location, is a common pattern of disease progression in EC. The LN metastasis rates for pathologic T1, T2, and T3 EC after surgery are 15–20, 40–50, and 70–75%, respectively (2, 3). Accordingly, surgery for thoracic EC frequently requires a meticulous LN dissection encompassing the cervico-thoracic-abdominal area in addition to subtotal or total esophagectomy. This extensive surgical procedure for EC can result in a postoperative mortality rate of 0–7% (4–6), and complications include laryngeal nerve palsy and gastrointestinal dysfunction (7, 8).

Radiotherapy (RT) with or without concurrent chemotherapy is often accepted for definitive treatment of T1 EC. The use of involved-field RT (IFRT) *versus* elective nodal irradiation (ENI) is controversially discussed, as skipped LN metastasis substantially occurs outside of the RT field boundary after IFRT, even with chemotherapy. Moreover, these regional recurrences are difficult to salvage, whereas most cases of local recurrence in the esophagus or metachronous primary EC are successfully salvaged with endoscopic resection or surgery (9–11). Therefore, the necessity of extended-field RT (EFRT) has been increasingly emphasized (12). We also previously reported a study of 24 patients with superficial EC presenting satisfactory outcomes after EFRT without chemotherapy targeting at least the entire esophagus (13).

Modern photon RT techniques including three-dimensional conformal RT (3D-CRT) and intensity-modulated RT (IMRT) have been widely used for EC treatment, but there are risks of acute or delayed cardiopulmonary toxicity due to substantial radiation exposure to the lung and the heart. Proton beam therapy (PBT) has a unique physical characteristic known as the Bragg peak, which allows the rapid elimination of a radiation dose after passing through the tumor target. PBT has been expected to reduce radiation-related toxicity effects by decreasing unintended exposure of healthy organs to radiation. Previous dosimetric studies have shown that in comparison to IMRT, PBT significantly decreases the radiation dose to the lung and heart (14, 15). Moreover, a recent study demonstrated that PBT significantly reduces the frequency of severe lymphopenia, which is known as a poor prognostic factor, compared to photon RT in neoadjuvant chemoradiation for EC (16).

In the present study, we compared treatment outcomes and toxicities of photon RT (3D-CRT and IMRT) with those of PBT and evaluated RT field issue in patients with T1–T3 thoracic EC without LN metastasis, the majority of whom underwent EFRT without chemotherapy.

## Materials and Methods

### Patients

The medical records of 77 patients with cT1–3N0M0 squamous cell carcinoma of thoracic esophagus who underwent definitive RT with or without chemotherapy at the National Cancer Center in Republic of Korea between November 2011 and November 2019 were retrospectively reviewed. Baseline characteristics included the medical history and physical examination results. Laboratory measurements included complete blood cell count and blood biochemistry measurements. For staging, esophagogastroduodenoscopy with Lugol staining, chest computed tomography (CT), endoscopic ultrasonography, and ^18^F-fluorodeoxyglucose positron emission tomography (FDG-PET) were performed. The clinical stage was based on the 7^th^ edition of the American Joint Committee on Cancer (AJCC) TNM classification. This study was approved by the Institutional Review Board of National Cancer Center in Republic of Korea (NCC2020-0043).

### Endoscopic Resection of T1 EC After Risk Assessment

Our multidisciplinary esophageal cancer oncology clinic by radiology, gastroenterology, radiation oncology, thoracic surgical oncology, medical oncology, and radiation oncology subspecialists recommends that some patients with T1 EC who underwent endoscopic submucosal dissection (ESD) in our hospital require further treatment with esophagectomy or RT with or without chemotherapy. Indications for further treatment are incompleteness of ESD including invasion of the submucosa or lymphovascular space by the tumor, or close or positive resection margins. Patients who were medically inoperable or those who were reluctant to undergo surgery after ESD for T1 EC were treated with RT, as were those treated without ESD for T1 EC.

### Radiotherapy

RT was delivered using megavoltage photon beams or proton beams. A conventional fractionation schedule with a fraction size of two cobalt gray equivalent (CGE) was used for all patients. The gross tumor volume (GTV) was defined as the tumor volume visualized on chest CT or FDG-PET or as the area marked with clips located near the proximal and distal ends of the primary tumor or ESD bed by a specialized upper gastrointestinal endoscopist before undergoing an RT simulation CT. In patients with esophageal clips, clip migration or removal was verified before the start of the RT.

The shrinking field technique was used for all patients. For IFRT, the initial clinical target volume (CTV2) extended 5 cm craniocaudally and 1–2 cm radially from the GTV. For EFRT, the CTV2 encompassed the entire esophagus and regional LN group including the pretracheal, retrotracheal, paratracheal, subcarinal, and peri-esophageal LNs with or without the supraclavicular LN (SCN) and a part of the abdomen including the paracardial, left gastric, and celiac LNs. The final clinical target volume (CTV1) covered the GTV with a margin of at least 1–2 cm craniocaudally and radially. Subsequently, the planning target volume (PTV) was defined as the CTV plus 0.5–1 cm. Total doses of 38–46 CGE (median 44 CGE) and 50–66 CGE (median 66 CGE) were delivered once daily to the initial PTV (PTV2) and the final PTV (PTV1), respectively.

The 3D-CRT and PBT plans were generated using the Eclipse^R^ planning system (Varian Medical System, Palo Alto, CA). During the 3D-CRT and PBT planning process, anterior-posterior parallel-opposed fields were usually selected in the initial phase to reduce the dose to the lungs, followed by anterior–posterior oblique fields or two posterior oblique fields in the boost phase to limit the dose to the spinal cord. For PBT, passive-scattering, uniform scanning, or pencil beam scanning techniques were used. The IMRT plan was generated using the Eclipse^R^ planning system or the Tomotherapy^R^ Planning System (Accuray, Sunnyvale, CA), and 3–5 fields were used with a dynamic multileaf collimator or helical tomotherapy unit. Photon beams of 6–15 MV and proton beams of 230 MeV were used.

### Chemotherapy

Capecitabine with or without platinum was the most commonly used regimen (n = 11), followed by bolus 5-fluorouracil (5-FU, n = 3) and paclitaxel-based chemotherapy (n = 3). Two cycles of 5-FU- or a capecitabine-based regimen with or without cisplatin were delivered starting on days 1 and 22. Capecitabine was administered orally twice daily for 14 days at a dose of 2,500 mg/m2/day. 5-FU was administered at 600–700 mg/m2/day on days 1–4 and days 22–25, and cisplatin was infused intravenously at 60–75 mg/m2 on days 1 and 22. Paclitaxel (50 mg/m2) and carboplatin [area under the curve (AUC) = 2] were administered weekly for 5–6 cycles during RT. No patient underwent neoadjuvant chemotherapy.

### Follow-Up and Statistical Analysis

Patients were examined weekly to evaluate acute treatment-related toxicities and the patients’ general condition during RT; they were then followed up with esophagogastroduodenoscopy or chest CT every 3 months for the first 2 years and every 6 months thereafter. Acute and chronic treatment-related toxicities were graded based on the Common Terminology Criteria for Adverse Events (CTCAE), version 4.0. Treatment responses were evaluated according to the Revised Response Evaluation Criteria in Solid Tumors (RECIST) Guidelines, version 1.1.

For the patterns of failure analysis, recurrences were subdivided into (1) local recurrence, progression of the primary tumor or occurrence of metachronous primary EC; (2) regional recurrence, spread to regional LNs based on the 7th edition of the AJCC TNM classification; and (3) distant LN or solid organ metastasis such as to the liver or lungs. Recurrences were also classified depending on their location as within (infield), at the boundary of (marginal), or outside (outfield) the initial RT field.

Survival duration was calculated from the first day of RT until the day of the last follow-up or the occurrence of events such as local failure, regional failure, distant failure, or death. The chi-square test or Fisher’s exact test was used to assess the measures of association between the risk factors and treatment-related toxicities as appropriate. The Wilcoxon rank-sum test was used to compare differences in continuous variables between two groups. Survival rates were calculated with the Kaplan–Meier method, and survival differences between groups were analyzed using the log-rank test. Statistical analyses were conducted using R Statistical Software version 4.0.3 (R Foundation for Statistical Computing, Vienna, Austria). The “gganatogram” package was used to visualize the sites of recurrences (17).

## Results

### Patient and Treatment Characteristics

The median age of all patients was 72 years (range, 44–89 years), with 70 (90.9%) men and seven (9.1%) women. Most patients (92.2%) had an Eastern Cooperative Oncology Group (ECOG) performance status score of 0–1. The tumors were in the upper, mid-, and lower thoracic esophagus in seven (9.1%), 34 (44.2%), and 36 (46.7%) patients, respectively. Most patients (n = 61, 79.2%) presented with T1 tumors, and eight (10.4%) and eight (10.4%) patients presented with T2 and T3 tumors, respectively. The median tumor size was 2.5 cm and ranged from 0.6 to 17 cm. Photon RT and PBT were administered to 29 (37.7%) and 48 (62.3%) patients, respectively. The baseline patient and tumor characteristics are summarized in **Table 1**.

**Table 1:** Patient characteristics.

Characteristics	All, No. (%)	Photon RT, No. (%)	PBT, No. (%)	*p* value
No. of patients	77 (100)	29 (37.7)	48 (62.3)	
Age (years)				
Median	72 (44–89)	73 (44–89)	69 (47–85)	0.203
≤69	33 (42.9)	9 (31.0)	24 (50.0)	0.164
≥70	44 (57.1)	20 (69.0)	24 (50.0)	
Sex				
Male	70 (90.9)	26 (89.7)	19 (91.7)	1.000
Female	7 (9.1)	3 (10.3)	4 (8.3)	
Performance status				
ECOG 0	33 (42.9)	9 (31.0%)	24 (50.0%)	0.263
ECOG 1	38 (49.3)	17 (58.6%)	21 (43.8%)	
ECOG 2	6 (7.8)	3 (10.3%)	3 (6.2%)	
Histologic grade				
WD	7 (9.1)	3 (10.3%)	4 (8.3%)	0.168
MD	41 (53.2)	17 (58.6%)	34 (70.8%)	
PD	16 (20.8)	6 (20.7%)	10 (20.8%)	
Unknown	3 (3.9)	3 (10.3%)	0 (0.0%)	
Tumor location				
Upper thoracic	7 (9.1)	3 (10.3%)	4 (8.3%)	0.023
Middle thoracic	34 (44.2)	18 (62.1%)	16 (33.3%)	
Lower thoracic	36 (46.7)	8 (27.6%)	28 (58.3%)	
T stage				
T1	61 (79.2)	21 (72.4%)	40 (83.3%)	0.713
T2	8 (10.4)	3 (10.3%)	5 (10.4%)	
T3	8 (10.4)	5 (17.2%)	3 (6.2%)	
Tumor size (cm)				
Median	2.5 (0.6–17)	2.5 (0.6–8)	2.75 (1.0–17.0)	0.869
<3	40 (51.9)	16 (55.2%)	24 (50.0%)	0.838
≥3	37 (48.1)	13 (44.8%)	24 (50.0%)	

RT, radiotherapy; PBT, proton beam therapy; ECOG, Eastern Cooperative Oncology Group; SCC, squamous cell carcinoma; WD, well differentiated; MD, moderately differentiated; PD, poorly differentiated.

The SCN and a part of the abdomen were irradiated in 53 (68.8%) and 44 (57.1%) patients, respectively. Abdominal LN irradiation was more frequently performed in the PBT group (72.9 *versus* 31.0%, *p* = 0.001). Twenty-six patients received RT after ESD due to submucosal invasion and close or involved resection with margins less than 1 mm. Concurrent chemotherapy was administered to five (8.2%) patients with T1 tumors and 12 (75.0%) patients with T2–3 tumors. Details about the treatments are summarized in **Table 2**. The percentage of patients who received chemotherapy did not differ between groups.

**Table 2:** Treatment characteristics.

Characteristics	All, No. (%)	Photon RT, No. (%)	PBT, No. (%)	*p* value
No. of patients	77 (100)	29 (37.7)	48 (62.3)	
Radiation dose				
Median, CGE	66 (50–66)	64 (56–66)	66 (50–66)	0.004
Radiation field				
Involved	10 (13.0)	5 (17.2)	5 (10.4)	0.489
Extended	67 (87.0)	24 (82.8)	43 (89.6)	
SCN irradiation				
Performed	53 (68.8)	22 (75.9)	31 (64.6)	0.435
Not performed	24 (31.2)	7 (24.1)	17 (35.4)	
Abdominal LN irradiation				
Performed	44 (57.1)	9 (31.0)	35 (72.9)	0.001
Not performed	33 (43.9)	20 (69.0)	13 (27.1)	
ESD				
Yes	26 (33.8)	12 (41.4)	14 (29.2)	0.396
No	51 (66.2)	17 (58.6)	34 (70.8)	
CCRT				
Performed	17 (22.0)	9 (31.0)	8 (16.7)	0.569
Not performed	60 (78.0)	20 (69)	40 (83.3)	
Chemotherapy regimen				
5-FU	1 (1.3)	1 (3.4)	0	0.250
5-FU/cisplatin	2 (2.6)	2 (6.9)	0	
Capecitabine	8 (10.4)	4 (13.8)	4 (8.3)	
Capecitabine/cisplatin	3 (3.9)	2 (6.9)	1 (2.1)	
Paclitaxel/carboplatin	3 (3.9)	0	3 (6.3)	

RT, radiotherapy; PBT, proton beam therapy; CGE, cobalt gray equivalent; SCN, supraclavicular lymph nodes; LN, lymph nodes; ESD, endoscopic submucosal dissection; CCRT, concurrent chemoradiotherapy; 5-FU, fluorouracil.

### Dosimetric Analysis

**Figure 1:**
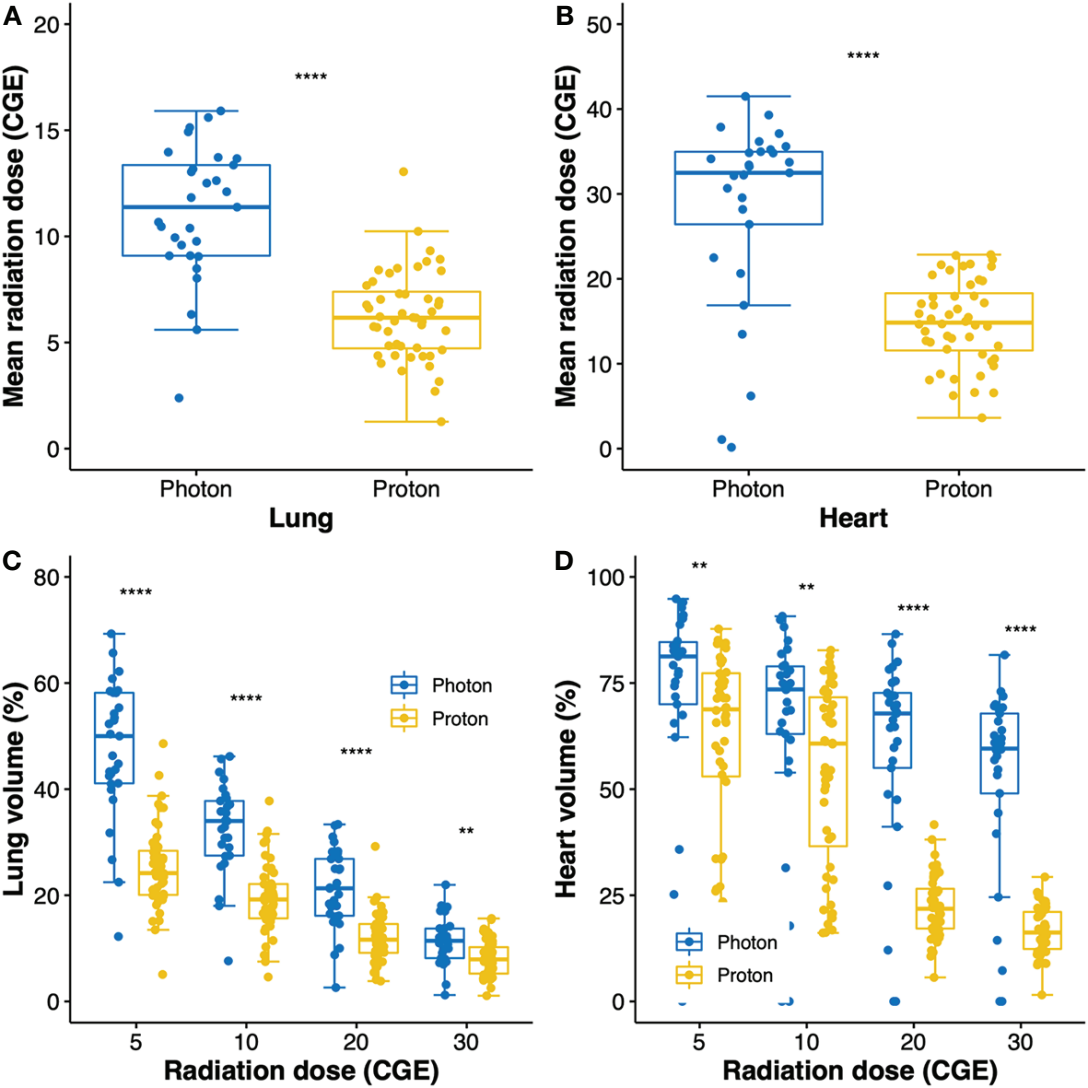
Box plots of mean radiation doses to the lung **(A)** and heart **(B)**. Box plots showing the distributions of dose–volume indices for the lung **(C)** and heart **(D)**. Whiskers indicate 1.5 times the interquartile range above and below the mean; dots represent individual observations. CGE, cobalt gray equivalent. **p < 0.01, ****p < 0.0001.

Radiation exposures to the lung and heart are compared between groups in **Figure 1**. Compared to photon RT, PBT significantly reduced the mean radiation doses to the lung (11.1 *versus* 6.3 CGE, respectively, *p* < 0.001; **Figure 1A**) and heart (32.5 *versus* 14.8 CGE, respectively, *p* < 0.001, **Figure 1B**). Moreover, the V doses (from V5 to V30) of the lung and heart were significantly lower in the PBT group than in the photon RT group (**Figures 1C, D**).

### Survival and Disease Control

**Figure 2:**
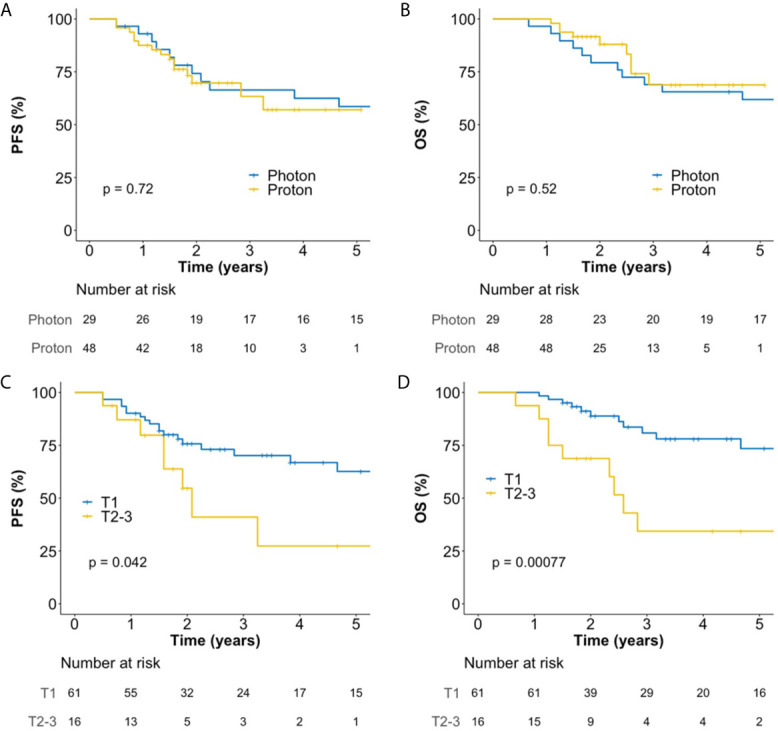
Kaplan–Meier plot of progression-free survival **(A)** and overall survival **(B)** rates according to the use of photon RT and PBT in all patients over 60 months. Kaplan–Meier plot of progression-free survival **(C)** and overall survival **(D)** rates according to T stage over 60 months. OS, overall survival; PBT, proton beam therapy; PFS, progression-free survival; RT, radiotherapy.

The median follow-up duration for all patients was 46 (interquartile range, 22–72) months, and those for patients treated with photon RT and PBT were 78 (interquartile range, 69–97) and 25 (interquartile range, 21–42) months, respectively. In all patients, the 5-year progression-free survival (PFS) and overall survival (OS) rates were 56.5 and 64.9%, respectively. There were no significant differences in PFS (**Figure 2A**) and OS (**Figure 2B**) between PBT and photon RT. In patients with T1 cancer, the 5-year PFS and OS rates were 62.6% (**Figure 2C**) and 73.5% (**Figure 2D**), respectively. During the follow-up period, a total of 22 deaths were observed. Among them, 13 were due to disease progression, and one was due to postoperative myocadiac infarction after salvage esophagectomy for local recurrence. The remaining eight were not related to esophageal cancer.

### Patterns of Failure Analysis

**Figure 3:**
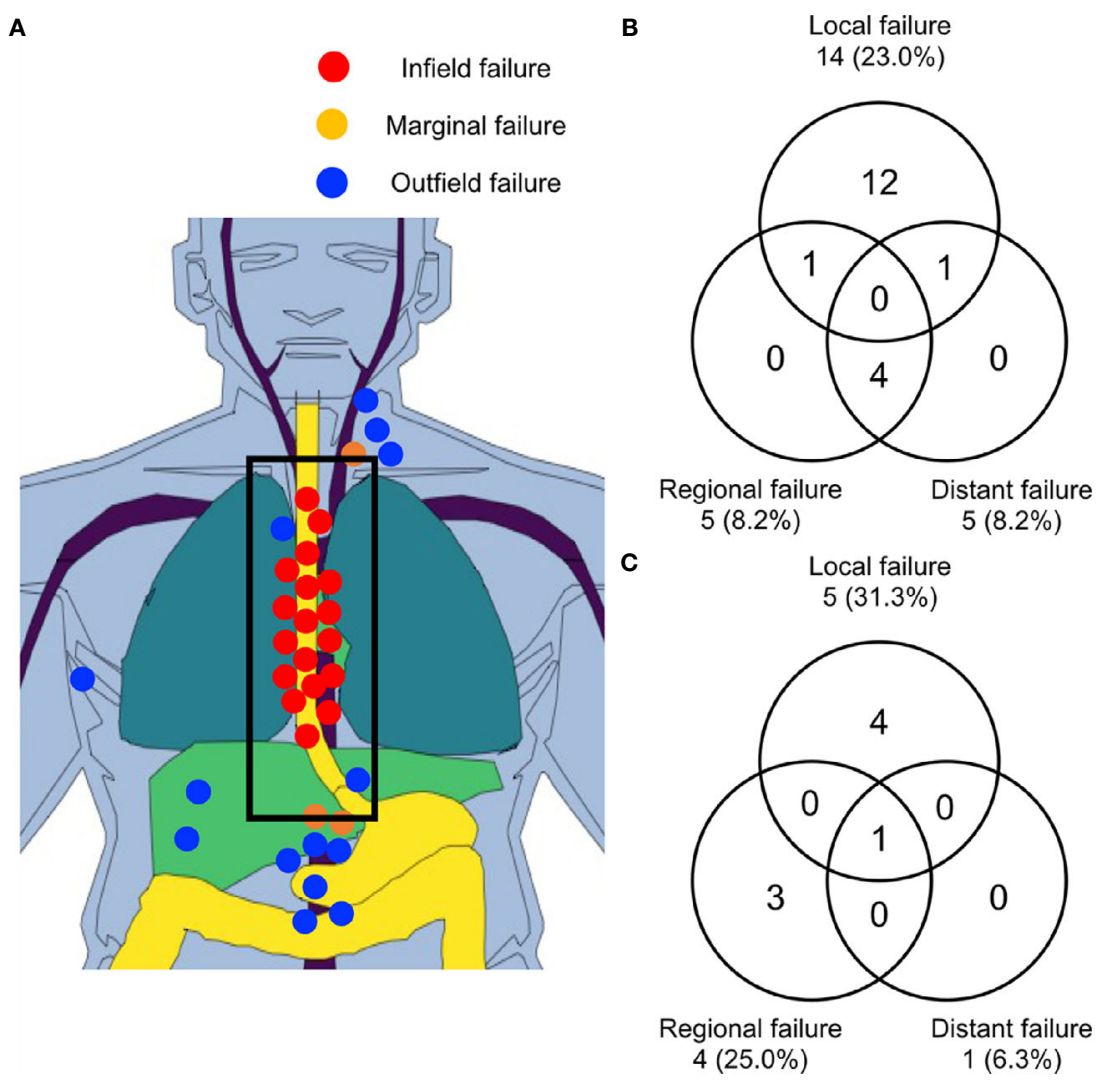
Failure sites according to the RT field in all patients **(A)**. Patterns of treatment failure for patients with T1 **(B)** and T2–3 **(C)** cancer. RT, radiotherapy.

Recurrences were observed in 34 sites of 26 patients. All infield recurrences occurred only in the esophagus (n = 19); we observed no infield LN metastasis. Among these recurrences, 17 were due to the progression of primary tumors, and two were caused by the development of metachronous primary esophageal cancers. There were three marginal recurrences, of which one and two were detected in the SCN and left gastric LN, respectively. Outfield failures were observed in 12 sites including the upper cervical LN (n = 1), SCN (n = 2), mediastinal LN (n = 2), celiac LN (n = 2), abdominal para-aortic LN (n = 2), liver (n = 2), and bone (n = 1). In the patient with upper cervical LN recurrence, the SCN was included in the RT field, but regional recurrences occurred at the boundary (SCN) and the outside (upper cervical LN) of the RT field. Two patients, who had received IFRT, experienced outfield mediastinal LN recurrence. In the two patients who presented with outfield celiac LN recurrence, the celiac LN was not included in the RT field. All solid organ metastases developed synchronously with LN metastasis or consecutively following preceding LN metastasis. The sites of recurrences according to the RT field are shown in **Figure 3A**. Local, regional, and distant failure rates according to the T stage are shown in **Figures 3B, C**. Local failure was most common, followed by regional and distant failures. No isolated distant metastasis was without local failure or LN metastasis.

**Figure 4:**
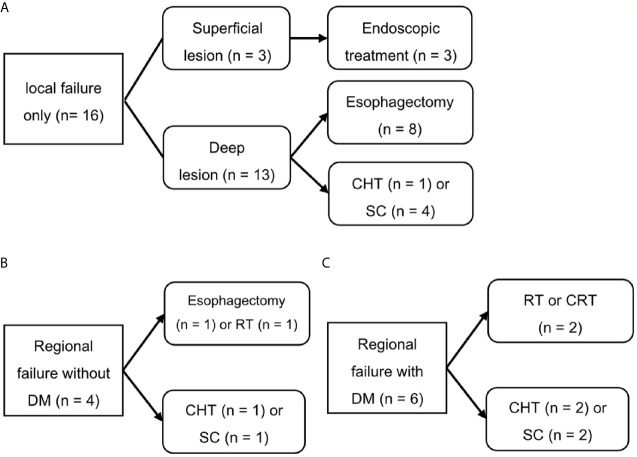
Salvage treatment scheme for patients who experienced isolated local recurrence **(A)**, regional recurrence without distant metastasis **(B)**, or distant metastasis **(C)**. SC, supportive care; CHT, chemotherapy; CRT, chemoradiotherapy; DM, distant metastasis; RT, radiotherapy.

**Figure 4** shows the salvage treatments according to failure patterns. Among the 16 patients who experienced isolated local recurrence without regional and distant metastasis, three patients with superficial recurrent cancers were treated endoscopically including argon plasma coagulation (n = 1) and ESD (n = 2), whereas the 13 patients with deep recurrent cancers underwent esophagectomy (n = 8), chemotherapy (n = 1), and supportive care (n = 4). The four patients who presented with regional recurrence without distant metastasis were treated with esophagectomy (n = 1), radiotherapy (n = 1), chemotherapy (n = 1), and supportive care (n = 1). Of the nine patients who underwent esophagectomy, one patient, who underwent photon RT, died due to postoperative myocadiac infarction, whereas the remaining eight patients (seven with PBT and one with photon RT) were alive and disease-free until the most recent follow-up. Consequently, among 16 patients with isolated local recurrence, 11 (68.8%) patients were salvaged with esophagectomy or endoscopic treatment. Regional recurrences were only observed at the boundary or outside of the radiotherapy field, never inside this field. However, their salvage rate was poor (20%).

### Treatment-Related Toxicities

Although almost all patients presented with acute esophagitis, most cases were grade 1 (70.1%). Grade ≥2 acute esophagitis developed significantly more in patients who underwent concurrent chemoradiotherapy (64.7%) than in those with RT alone (20%, *p* = 0.001). Pleural and pericardial effusion developed in 20 (26.0%) and 11 (14.3%) patients, respectively. Among those, only one photon RT patient, who had a history of paroxysmal atrial fibrillation, required hospitalization owing to pericardial effusion after the completion of concurrent chemoradiotherapy. Among all other patients, no patient presented with grade ≥3 pleural or pericardial effusion. Radiation pneumonitis was observed in 55 patients (71.4%). However, except for one case with grade 2 radiation pneumonitis, all patients presented with grade 1 radiation pneumonitis. The incident rates of these complications did not differ between the photon RT and PBT groups.

## Discussion

The strategy to reduce RT-related cardiopulmonary toxicity in EC has recently been more emphasized since trimodal therapies like neoadjuvant chemoradiation followed by surgery have been established as a popular standard treatment regimen in most of the operable EC cases (18, 19) Therefore, efforts to reduce the radiation dose to organs at risk, especially the lung and the heart, in EC have been made by applying modern RT techniques. As an advanced photon RT technique, IMRT has already shown superior capabilities in comparison to 3D-CRT to decrease radiation exposure to organs at risk in EC at various locations (20–23), and its application has been associated with significantly higher OS compared to 3D-CRT by reducing cardiac-related deaths (24). PBT has been spotlighted to further reduce the incidence of RT-associated cardiopulmonary complications due to its superior physical properties in comparison to photon RT. Dosimetric studies have already demonstrated that PBT significantly reduces radiation to the whole heart and its substructures, including the left anterior descending artery, left ventricle, and pericardium, compared to 3D-CRT and IMRT (25, 26). In a retrospective study, postoperative pulmonary and GI complication rates were significantly reduced or had the trend towards fewer complications in patients with PBT compared to those with 3D-CRT or IMRT, suggesting that the radiation modality can be an important factor to reduce complication rates in trimodal EC therapy (27).

The present study evaluated treatment outcomes and toxicities of photon RT and PBT for thoracic T1–3 EC without LN metastasis. In the dosimetric comparative analysis, significantly lower radiation doses to organs at risk including the lung and heart were observed in the PBT group compared to the photon RT group. In a previous phase II study comparing IMRT and PBT in definitive or neoadjuvant concurrent chemoradiotherapy for stage II–III esophageal cancer, PBT showed reduced toxicities, but similar PFS compared with IMRT (28). However, in the present study, OS, PFS, and RT-related toxicities were not significantly different between the photon RT and PBT groups. Although our data did not demonstrate clinical advantages of PBT in terms of clinical outcomes and treatment-related toxicities, the benefits of PBT might be difficult to prove in the current study for the following reasons. The overall toxicity effects in both photon RT and PBT groups seemed to be far from severe, possibly because most of the patients (78%) did not receive concurrent chemotherapy. Moreover, a rather long-term follow-up might be needed to evaluate delayed cardiopulmonary toxicity in patients with higher life expectancy, with 79.2% of all patients being classified as T1. In the previous phase II study, all patients received concurrent chemotherapy, and about half of patients underwent surgery after chemoradiation. These might contribute to higher adverse event rates than our study and evident clinical benefit of PBT in reducing treatment-related toxicities. In the present study, among the patients treated with esophagectomy due to local recurrence, one case of postoperative mortality was observed in the photon RT group, whereas all seven patients treated with PBT successfully underwent salvage esophagectomy without serious postoperative morbidity. As suggested by another study (27, 28), PBT might reduce morbidities associated with salvage or planned esophagectomy following neoadjuvant RT, but this is beyond the scope of our analysis.

We also evaluated the effects of the radiation field for T1–3 thoracic EC without LN metastasis in the current study. Notably, EFRT provided good disease control with acceptable toxicity profiles in patients of both photon RT and PBT groups. Although most of the T1 EC patients received EFRT without chemotherapy (90.2%), their 5-year OS rate was 73.5% which is comparable to rates previously reported for surgical outcomes (29–32). As there was no clear guideline for RT in T1 EC, we designed our own treatment scheme including the radiation dose, field, and the use of concurrent chemotherapy on the basis of the following hypotheses: 1) a total radiation dose of 64–66 Gy without chemotherapy would be sufficient for most of the superficial lesions, similar to the dose for T1 hypopharyngeal cancer; and 2) the elective radiation dose would eradicate clinically invisible LN metastases, similar to the effects observed in hypopharyngeal cancer.

For T1 EC, concurrent chemoradiotherapy is generally considered when esophagectomy is not applicable. Nemoto et al. reported that RT with or without concurrent chemotherapy resulted in 3-year OS rates of 90 and 70% for mucosal cancer (T1a) and submucosal cancer (T1b), respectively. OS of patients treated with local RT and concurrent chemotherapy was slightly, although not significantly, better than that of patients treated with ENI without chemotherapy (33). Koide et al. analyzed the treatment outcomes of 60 Gy IFRT combined with concurrent chemotherapy in patients with T1 EC. In this population, IFRT of 60 Gy with concurrent chemotherapy resulted in 5-year OS, PFS, and local control rates of 77.0, 46.9, and 62.7%, respectively (34). The results of these studies suggest that our outcomes of EFRT without chemotherapy are comparable to those of IFRT with concurrent chemotherapy.

Motoori et al. reported the treatment outcomes of radical esophagectomy and definitive chemoradiotherapy for clinical T1bN0M0 EC (35). They showed that local recurrences after definitive chemoradiotherapy could be controlled with salvage esophagectomy, as observed in all three cases with local recurrence. However, among 20 patients with all types of recurrence, the prognosis of 13 (65%) patients with LN recurrence was poor after definitive chemoradiotherapy. Only four of these 13 cases could be salvaged with LN dissection or chemoradiotherapy. In our study, 12 patients of the 16 patients with isolated local recurrence could be salvaged with esophagectomy or endoscopic treatment, although one patient treated with photon RT died due to postoperative myocardial infarction. Another case with synchronous local and regional LN recurrence in the left gastric LN was also successfully salvaged by esophagectomy. In our study, regional recurrences were only observed at the boundary or outside of the RT field, never inside the radiation field. Furthermore, all cases of distant LN or solid organ metastasis were detected synchronously with or after the development of regional recurrences (**Figures 3B, C**). These results indicate that regional control is very important to improve OS in patients with LN-negative EC. This also supports the use of ENI to control subclinical regional diseases within the radiation field.

Although several retrospective studies have demonstrated that concurrent chemoradiotherapy leads to acceptable OS and PFS rates, no prospective study has answered yet the question of whether concurrent chemotherapy is essential, nor has any study compared the efficacy of IFRT and EFRT for T1 EC. Historically, concurrent chemotherapy increased the incidence of severe acute toxic effects, as suggested in the RTOG 85-01 study (36). In the current study, EFRT without chemotherapy showed satisfactory treatment outcomes, especially in T1 EC, and acceptable toxicities in the lung and esophagus, whereas the use of chemotherapy tended to increase the risk for grade ≥2 acute esophagitis. This suggests that EFRT without chemotherapy can be considered a treatment option for T1 EC. Esophagectomy is the current standard treatment for patients with lymphovascular or submucosal invasion after ESD. In our study, treatment outcomes were superior in T1 patients who underwent ESD compared to those who did not. These results may be due to a reduced tumor burden after ESD, and/or as an earlier clinical stage without submucosal invasion for patients who underwent ESD. These data indicate that our treatment strategy is applicable to non-curative endoscopic resections.

The survival outcomes of patients with locally advanced EC after definitive chemoradiation are still not satisfactory. Our study confirmed this even in LN-negative EC with mainly local progression, the predominant pattern of failure. Interestingly, this characteristic pattern of lymphatic metastasis was also found in T2–3N0 EC similar to that seen in T1 EC, with a percentage of patients presenting LN metastasis and distant metastasis beyond the RT boundary despite the primary tumor being well-controlled. This finding might suggest the necessity of ENI in T2–3N0 EC. Although the importance of regional control in EC was traditionally emphasized in surgical series (29, 32, 37), the necessity of ENI has also been discussed in definitive RT for T1 EC, as well as in more advanced EC (12, 13). A Japanese study group reported promising results of definitive chemoradiation with ENI for stage II–III EC (12). However, hematological or other ENI-associated toxicities might impede the adoption of ENI. In this respect, PBT can be a useful tool to minimize treatment-related toxicities while maintaining the advantage of ENI in the definitive or neoadjuvant CRT setting. PBT may provide even additional benefits related to the reduction in hemato-immunological toxicity. In the propensity score-matched analysis between neoadjuvant PBT and IMRT for EC, patients with PBT developed markedly less frequently grade 4 lymphopenia than those with IMRT, and PBT was found to be an independent predictor of grade 4 lymphopenia, which only trended for poor OS but significantly reduced PFS (16). Other recent studies also demonstrated that lymphopenia is an adverse prognostic factor after chemoradiation in various cancers including EC (38, 39) These findings indicate that PBT can improve the survival of EC patients by reducing late treatment-related toxicities, as well as radiation-induced lymphopenia.

The limitation of our study are its retrospective and non-randomized nature and the small number of enrolled patients, because esophagectomy is the standard treatment for T1 EC. In addition, we could not evaluate the patients’ quality of life after RT, and the follow-up duration for the PBT group was shorter than that for the photon RT group. Currently, we are conducting a prospective single-arm study at our institution to investigate the efficacy of wider extended-field PBT including almost all lymphatic channels for T1 EC without chemotherapy (Clinical Research Information Service, Korea Centers for Disease Control and Prevention; KCT0004853).

In conclusion, the dosimetric benefit of significantly reduced radiation doses to the lung and heart in the PBT group compared to the photon RT group did not result in a benefit regarding survival outcome and clinical toxicity in T1–3 EC without LN metastasis. Furthermore, our data demonstrated that EFRT using photon RT and PBT resulted in satisfactory outcomes in patients with T1 EC. Because the characteristics of the lymphatic disease dissemination in T2–3N0 EC were similar to those in superficial EC, we think that EFRT deserves an in-depth investigation regarding locoregional control and overall toxicity in patients with localized EC.

## Data Availability Statement

The raw data supporting the conclusions of this article will be made available by the authors, without undue reservation.

## Ethics Statement

The studies involving human participants were reviewed and approved by the Institutional Review Board of National Cancer Center in Republic of Korea (NCC2020-0043). Written informed consent for participation was not required for this study in accordance with the national legislation and the institutional requirements.

## Author Contributions

Y-GS was responsible for data contribution, data collection, statistical analysis, and manuscript editing. UB was responsible for data collection. HK, JL, MK, and YL were responsible for data contribution. DL was responsible for data collection. SL and TK were responsible for data interpretation. SM was responsible for study design, data contribution, data collection, data interpretation, writing of the manuscript, and all manuscript revisions, and should be considered the guarantor for the article as a whole. All authors contributed to the article and approved the submitted version.

## Funding

This study was supported by grants from National Cancer Center Grant (NCC 2110350-1) and the Korea Health Technology R&D Project through the Korea Health Industry Development Institute (KHIDI) funded by Ministry of Health and Welfare, Republic of Korea (HC19C0293). The funding source had no role in study design, data curation, or analysis and interpretation of the data.

## Conflict of Interest

The authors declare that the research was conducted in the absence of any commercial or financial relationships that could be construed as a potential conflict of interest.
